# Association of Frailty With the Risk of Mortality and Resource Utilization in Elderly Patients in Intensive Care Units: A Meta-Analysis

**DOI:** 10.3389/fmed.2021.637446

**Published:** 2021-10-04

**Authors:** Feiping Xia, Jing Zhang, Shanshan Meng, Haibo Qiu, Fengmei Guo

**Affiliations:** ^1^School of Medicine, Southeast University, Nanjing, China; ^2^Jiangsu Provincial Key Laboratory of Critical Care Medicine, Department of Critical Care Medicine, Zhongda Hospital, School of Medicine, Southeast University, Nanjing, China

**Keywords:** frailty, elderly intensive care unit patients, mortality, hospital length of stay, meta-analysis

## Abstract

**Background:** The associations of frailty with the risk of mortality and resource utilization in the elderly patients admitted to intensive care unit (ICU) remain unclear. To address these issues, we performed a meta-analysis to determine whether frailty is associated with adverse outcomes and increased resource utilization in elderly patients admitted to the ICU.

**Methods:** We searched PubMed, EMBASE, ScienceDirect, and Cochrane Central Register of Controlled Trials through August 2021 to identify the relevant studies that investigated frailty in elderly (≥ 65 years old) patients admitted to an ICU and compared outcomes and resource utilization between frail and non-frail patients. The primary outcome was mortality. We also investigated the prevalence of frailty and the impact of frailty on the health resource utilization, such as hospital length of stay (LOS) and resource utilization of ICU.

**Results:** A total of 13 observational studies enrolling 64,279 participants (28,951 frail and 35,328 non-frail) were finally included. Frailty was associated with an increased risk of short-term mortality (10 studies, relative risk [RR]: 1.70; 95% CI: 1.45–1.98), in-hospital mortality (five studies, RR: 1.73; 95% CI: 1.55–1.93), and long-term mortality (six studies, RR: 1.86; 95% CI: 1.44–2.42). Subgroup analysis showed that retrospective studies identified a stronger correlation between frailty and hospital LOS (three studies, MD 1.14 d; 95% CI: 0.92–1.36).

**Conclusions:** Frailty is common in the elderly patients admitted to ICU, and is associated with increased mortality and prolonged hospital LOS.

**Trial registration:** This study was registered in the PROSPERO database (CRD42020207242).

## Introduction

With the aging of the population, the number of elderly patients admitted to the intensive care unit (ICU) has been growing (1). Recently, approximately 20% of the ICU admissions are elderly patients, and this proportion is expected to increase in the next decade (2). During hospitalization in the ICU, elderly patients are considered to be more vulnerable to the stress induced by acute illnesses, since they have age-related physiological changes, are more likely to have chronic diseases and have a higher prevalence of frailty. In the context of the rational allocation of medical resources, especially during the COVID-19 epidemic, appropriate intensive care resource utilization is essential, and many physicians have doubts if elderly patients are benefitted from the ICU admission. It is challenging to identify elderly patients who may benefit from intensive treatment.

The concept of frailty originated in the field of geriatrics and has been introduced to critical care medicine. Frailty is used to describe a biological syndrome or state associated with aging that is characterized by decreased functioning of multiple physiological systems, accompanied by an increased vulnerability to stress (3). Characteristic physiologic and molecular features, such as increased oxidative stress and inflammatory markers, are observed in frail individuals (4–6). For the frail individuals, functional aging represented by frailty is more important than biological aging (7), and there is emerging evidence that frail individuals are more vulnerable to adverse outcomes and increased resource utilization across different disease states (8–17). Frailty has been indicated proven to be associated with increased mortality but not increased service utilization in patients who were critically ill (18). However, some results have been controversial concerning the elderly patients admitted to ICU, who are more vulnerable to frailty (19, 20). Thus, it is crucial to investigate the impact of frailty on the elderly patients admitted to ICU.

In this study, we conducted a meta-analysis to assess whether frailty in elderly ICU patients is predictive of adverse outcomes and increased resource utilization. We hypothesized that frailty was associated with an increased mortality and resource utilization in the elderly patients admitted to ICU.

## Methods

### Protocol and Registration

Our study was reported according to the Meta-analysis Of Observational Studies Epidemiology (MOOSE) guidelines (21), and the protocol was registered in PROSPERO (CRD42020207242).

### Information Sources and Searches

We initially searched electronic databases, including PubMed and EMBASE, in February 2020. Our search used keywords including “frailty” OR “frail” OR “frail elderly” AND “intensive care” OR “intensive care unit” OR “critical care” OR “critically ill” OR “critical ill” OR “critical illness.” The reference lists of selected articles were searched manually to identify additional studies. The literature search was updated in August 2021, adding the other research electronic databases of ScienceDirect, Cochrane Central Register of Controlled Trials.

### Study Selection

Two authors (FPX and JZ) carried out the literature search independently. We first removed duplicate records and then screened the titles and abstracts of all the articles for potential relevance. Records were identified as included, uncertain, or excluded. For uncertain records, the full text of the article was further investigated to determine its eligibility. The inclusion criteria were as follows: i) participants: elderly (every individual ≥65 years old) patients admitted to ICU; ii) exposure: frailty; iii) outcome: mortality or resource utilization; and iv) study design: prospective or retrospective cohort studies. We resolved disagreements by the discussion.

### Data Extraction

A data extraction sheet was developed in Excel to collect the following data: author, year, study design, country, frailty identification method, the sample sizes of frail, and non-frail patients, and the outcomes of interest. We chose outcomes that indicated the mortality of the patients and health services utilization. Outcomes maximally adjusted for available covariates were collected in our meta-analysis. The primary outcome was mortality, including short-term mortality (≤ 1 month after ICU admission), in-hospital mortality, and long-term mortality (≥6 months after ICU admission). Secondary outcomes were focused on health resource utilization, including hospital length of stay (LOS), use of mechanical ventilation, use of vasoactive agents, and use of renal replacement therapy. These data were independently extracted by FPX and JZ and later checked by SSM.

### Quality Assessment

We used the Newcastle–Ottawa Scale (NOS) to evaluate the quality of the studies included (22). The NOS is a validated scale for assessing the quality of observational studies, and it has the following three domains: selection of the study groups, comparability of the groups, and assessment of the outcomes. The NOS is a 9-point scale awarding a maximum of four stars for selection, two stars for comparability, and three stars for outcomes. Studies scoring 0–3 were deemed low quality; those scoring 4–6 were considered to be of moderate quality; and those scoring 7–9 were classified as high quality.

### Statistical Analysis

We calculated the relative risk (RR) with the corresponding 95% CI for mortality, use of mechanical ventilation, use of vasoactive agents, and use of renal replacement therapy with a random-effects model. In our meta-analysis, the RR was considered to be equivalent to the hazard ratio and the odds ratio (OR) (23). The weighted mean difference with 95% CI was calculated for the hospital LOS. We converted data to means and SDs when they were reported as medians (24). We conducted subgroup analyses with stratification by study type, age, frailty measure, and adjustment for confounders. Statistical heterogeneity among studies was determined with the Mantel–Haenszel (M–H) chi-squared test and the *I*^2^ statistic. Significant heterogeneity was defined as *I*^2^ value greater than 50% (25). An unadjusted, two-sided *p* < 0.05 was considered statistically significant. We performed the analyses using Review Manager 5.3 software (The Cochrane Collaboration, Copenhagen Denmark).

## Results

### Study Selection

**Figure 1:**
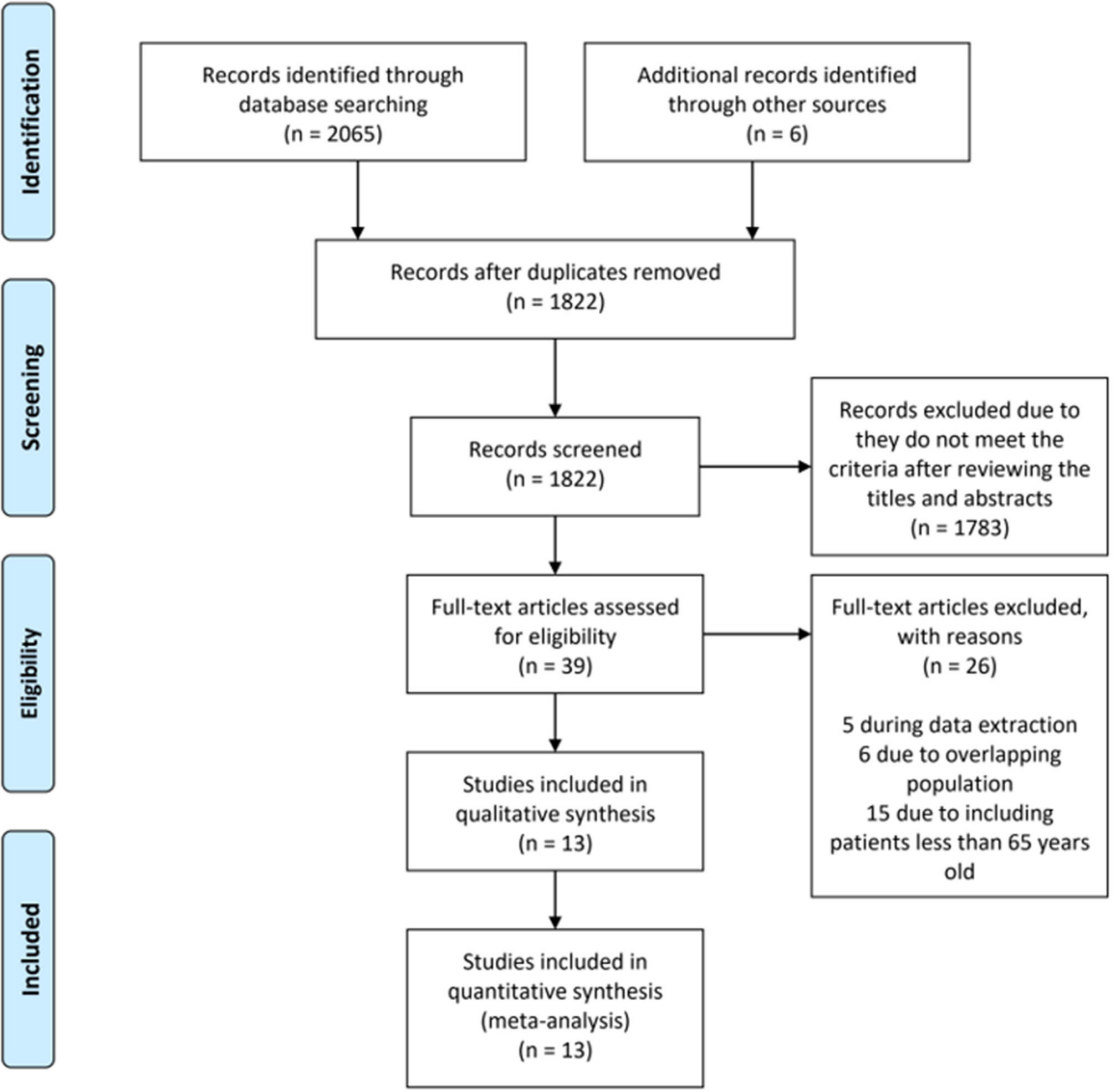
Preferred reporting items for systematic reviews and meta-analyses flow diagram.

The initial search identified 2,071 articles and abstracts. After the removal of duplicate articles, 1,822 remained. A further 1,783 records were excluded because they did not meet the criteria after the titles and abstracts were reviewed. A total of 39 full-text articles were assessed in detail. According to the inclusion criteria, 26 studies were excluded, leaving 13 studies (19, 20, 26–36) that were eligible for inclusion in the meta-analysis (Figure 1).

### Study Characteristics

**Figure 2:**
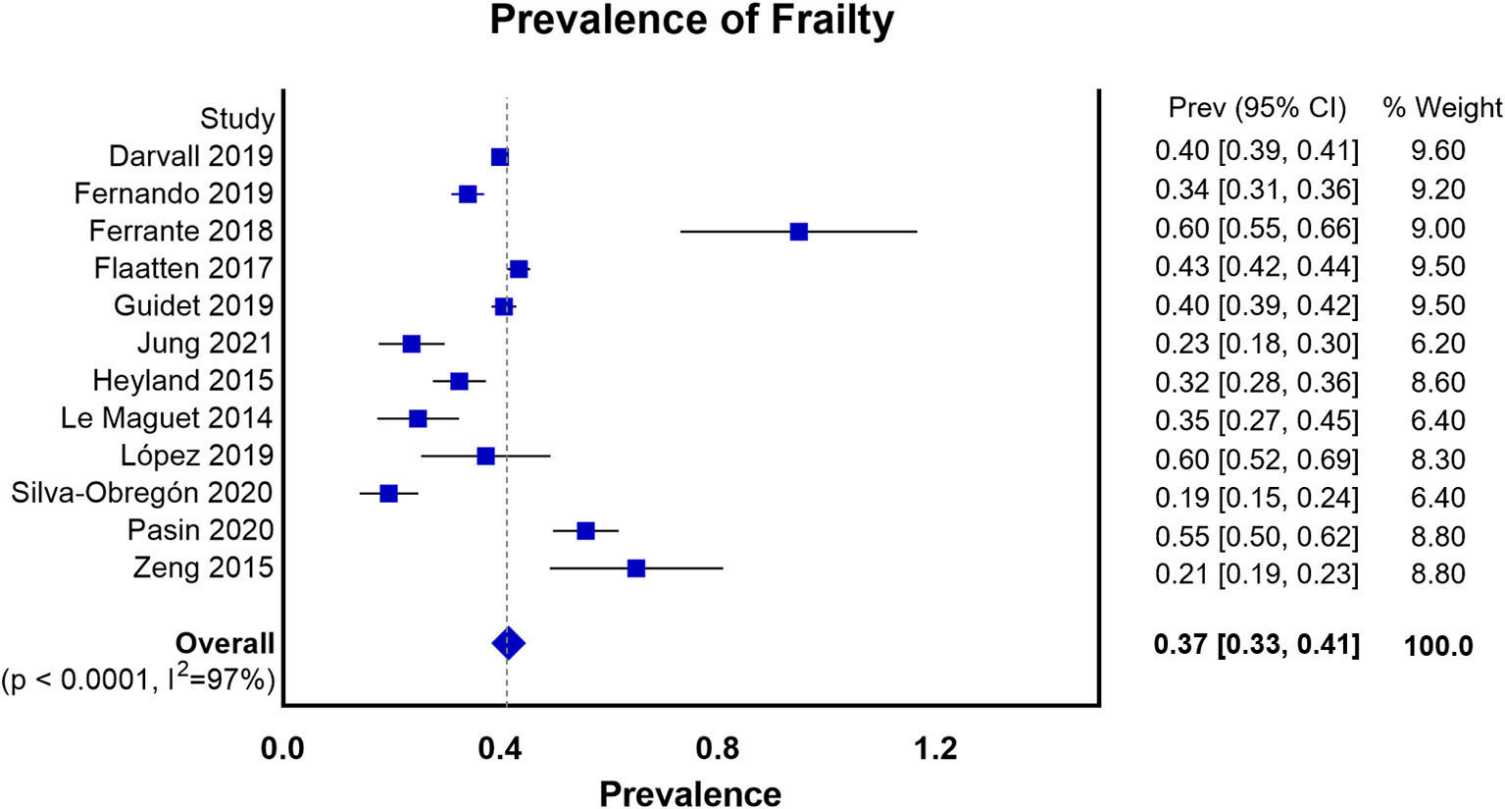
Prevalence of frailty in the elderly patients admitted to ICU according to all the measures of frailty.

We summarized the characteristics of the included studies in Table 1. The studies in our meta-analysis were published between 2014 and 2021. Nine of them were prospective observational studies, and the remaining four were retrospective cohort studies. Our meta-analysis enrolled 64,279 participants. Among them, 28,951 patients were frail, and 35,328 patients were classified as non-frail. The pooled data showed that the prevalence of frailty in the elderly population admitted to ICU studied was 0.37 (0.33, 0.41) (Figure 2). Nine studies assessed frailty with the clinical frailty scale (CFS) (37), two used the frailty index (FI) (38), one used the modified frailty index (mFI) (29), and one study used both the CFS and the frailty phenotype (FP) (39). The quality of the included studies ranged from 6 to 9 stars on the NOS, denoting that the studies were of high or moderate quality (Supplementary File 1).

**Table 1:** Characteristics of included studies.

**Author**	**Study design**	**Country**	**Sample size**	**Frailty [*n*(%)]**	**Age (years)**	**Frailty definition**	**Outcomes assessed**	**Variables adjustment of adjusted mortality reported**
Darvall et al. (19)	Retrospective cohort	Australia and New Zealand	15613	6203(39.7%)	84.6 ± 4.2	CFS > 4	Mortality/morbidity/ health service utilization	Sex, region, hospital type, and severity of illness
Fernando et al. (20)	Prospective cohort	Canada	1510	507(33.6%)	75.4 ±7.4	CFS > 4	Mortality/morbidity/ health service utilization	Age, sex, MODS, origin from long-term care, and comorbidity
Ferrante et al. (26)	Prospective cohort	USA	353	213(60.3%)	85.2 ±5.2	FI ≥ 3	Mortality/morbidity/ health service utilization	Age, gender, SOFA score, type of ICU admission
Flaatten et al. (27)	Prospective cohort	21 European countries	5021	2156(42.9%)	84.0 ± 3.7	CFS > 4	Mortality/morbidity	Age, gender, SOFA score, type of ICU admission
Guidet et al. (28)	Prospective cohort	22 European countries	3903	1568(40.2%)	84.0 ± 4.4	CFS > 5	Mortality/morbidity	Age, habitat, SOFA score, CPS and CFS
Hamidi et al. (29)	Retrospective cohort	USA	34854	17427(50.0%)	76.7 ± 7.0	NA	Mortality/morbidity/ health service utilization	NA
Heyland et al. (30)	Prospective cohort	Canada	609	193(31.7%)	85.0 ± 3.0	CFS > 4	Mortality/morbidity/ health service utilization	NA
Jung et al. (35)	Prospective cohort	28 countries	1346	279(20.7%)	75.0 ± 4.4	CFS ≥ 5	Mortality/morbidity/ health service utilization	Age, sex, comorbidities, SOFA score, BMI, PaO_2_/FiO_2_
Le Maguet et al. (31)	Prospective cohort	France	196	46(23.5%)	75.0 ± 6.0	FP>2; CFS > 4	Mortality/morbidity/ health service utilization	Sex, brain injury, SAPS II, glasgow coma scale, memory disorders, severe sepsis, septic shock, dialysis, ARDS, corticosteroid treatment
López et al. (32)	Prospective cohort	Spain	132	46(34.8%)	78.7 ± 6.7	CFS > 4	Mortality/morbidity/ health service utilization	APACHE II
Pasin et al. (36)	Retrospective cohort	Italy	302	167(55.3%)	84.0 ± 3.70	CFS ≥ 5	Mortality/morbidity/ health service utilization	Age, gender, BMI, CFS, MAP, chronic condition, cause of admission, need for suport treatment
Silva-Obregón et al. (33)	Retrospective cohort	Spain	285	53(18.6%)	77.6 ± 4.1	CFS > 5	Mortality/morbidity/ health service utilization	Gender, number of comorbidities, severity scores, treatment intensity and complications
Zeng et al. (34)	Prospective cohort	China	155	93(60.0%)	82.7 ± 7.1	FI > 0.22	Mortality/morbidity/ health service utilization	Age, sex

*CFS, clinical frailty scale; FI, frailty index; FP, frailty physical phenotype; ICU, intensive care unit; NA, not available*.

### Mortality

**Figure 3:**
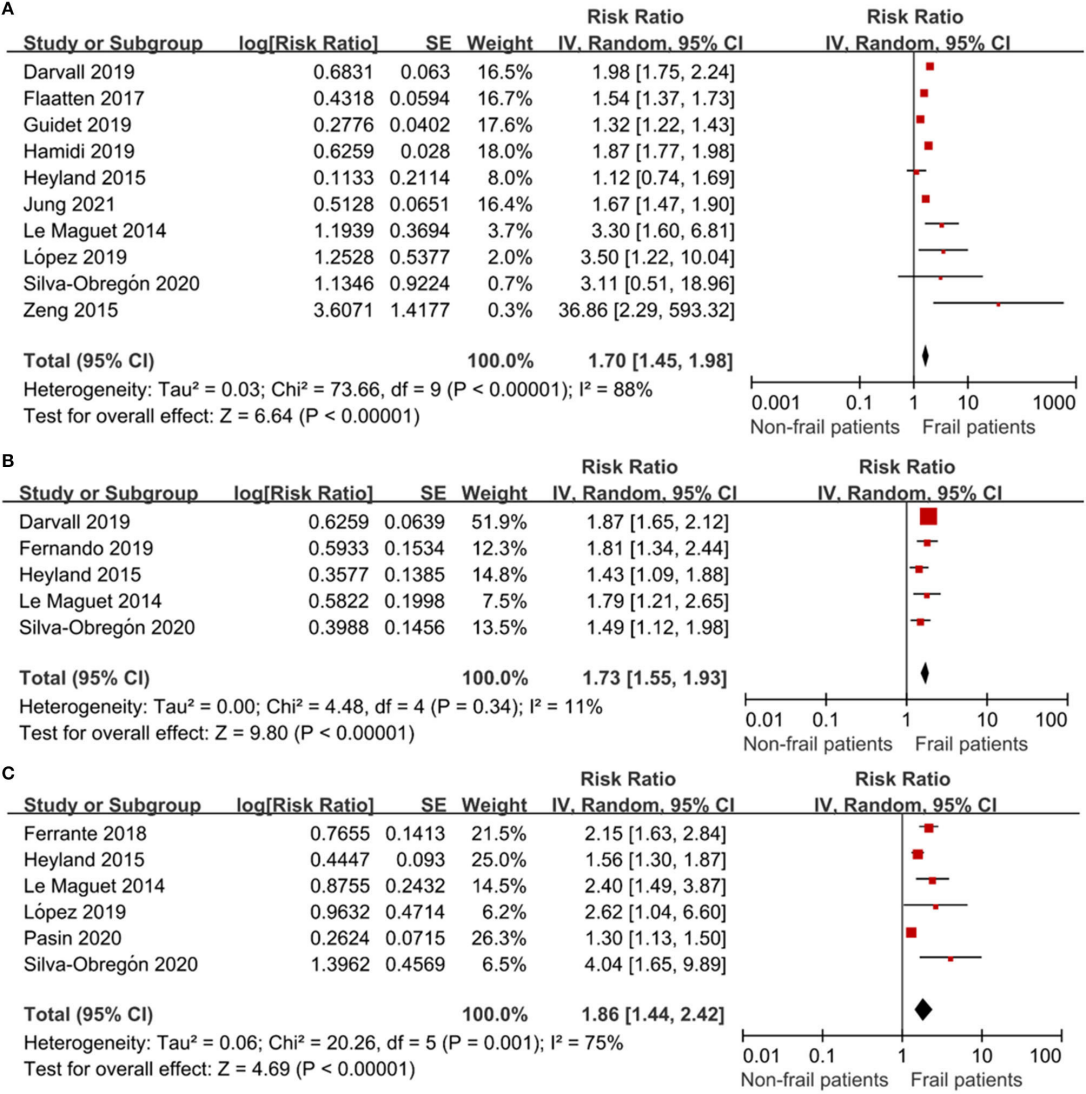
The association of frailty and mortality in elderly patients admitted to ICU. RR = relative risk. **(A)** The association of frailty and short-term mortality in elderly patients admitted to ICU. **(B)** The association of frailty and in-hospital mortality in the elderly patients admitted to ICU. **(C)** The association of frailty and long-term mortality in the elderly patients admitted to ICU.

All 13 studies reported the association between frailty and the risk of mortality. We extracted hospital mortality data from five studies, short-term mortality from 10 studies, and long-term mortality from six studies. The pooled unadjusted data revealed that frailty was associated with increased short-term mortality (RR: 1.70; 95% CI: 1.45–1.98; *I*^2^ = 88.0%; Figure 3A), in-hospital mortality (RR: 1.73; 95% CI: 1.55–1.93; *I*^2^ = 11.0%; Figure 3B), and long-term mortality (RR: 1.86; 95% CI: 1.44–2.42; I2 = 75.0%; Figure 3C). Eleven studies reported outcomes adjusted for different covariates, including age, sex, region, hospital type, severity of illness, treatment, and type of ICU admission (Table 1).

Subgroup analysis was conducted to determine the association of frailty with short-term and long-term mortality. The results showed that neither short-term nor long-term mortality was significantly affected by study location, age, the frailty measure, or adjustment for confounders (Table 2).

**Table 2:** Subgroup analysis on the association between frailty and mortality.

**Variable**	**Short-term mortality**				**Long-term mortality**			
	**N**	**RR (95% CI)**	***I^**2**^* (%)**	***Pheterogeneity***	**N**	**RR (95% CI)**	***I^**2**^* (%)**	***Pheterogeneity***
**Research type**								
Retrospective	3	1.89 [1.80, 1.99]	0	0.61	2	2.09 [0.70, 6.27]	83	0.01
Prospective	7	1.56 [1.31, 1.86]	77	<0.01	4	1.94 [1.51, 2.49]	50	0.11
**Age**								
>65 years old	5	1.84 [1.62, 2.09]	41	0.15	3	2.68 [1.83, 3.93]	0	0.60
>80 years old	5	1.54 [1.23, 1.92]	89	<0.01	3	1.60 [1.24, 2.06]	81	<0.01
**Frailty measure**								
CFS	7	1.59 [1.34, 1.88]	85	<0.01	5	1.77 [1.34, 2.34]	59	<0.01
Other	3	2.88 [1.29, 6.43]	70	0.03	1	2.15 [1.63, 2.84]	Not applicable	Not applicable
**Adjustment for confounders**								
Severity scores Yes	6	1.55 [1.33, 1.81]	70	<0.01	4	2.32 [1.85, 2.90]	0	0.60
No	4	1.81 [1.51, 2.16]	73	0.01	2	1.41 [1.18, 1.68]	59	0.12

*CFS, clinical frailty scale; RR, relative risk*.

### Resource Utilization

Six studies reported the hospital LOS. The pooled results showed that frail and nonfrail patients did not have significantly different hospital LOSs (MD 1.52 days; 95% CI−0.40–3.43, *p* < 0.001, *I*^2^ = 93%; Supplementary File 2). A subgroup analysis was conducted for the study type, and frailty was associated with a longer LOS (MD 1.14 days; 95% CI: 0.92–1.36, *p* = 0.63, *I*^2^ = 0%) in the retrospective studies. In the three prospective studies, the MD for short-term mortality was 1.76 d; 95% CI−1.94–5.46; *p* = 0.06, *I*^2^ = 65% (Supplementary File 2).

Seven of the 13 studies compared the use of mechanical ventilation. There was no difference in the use of mechanical ventilation between frail and non-frail patients (RR: 0.91; 95% CI 0.80–1.04; *p* < 0.001; *I*^2^ = 80%; Supplementary File 3). In addition, five of the 13 studies reported the use of vasoactive therapy between frail and non-frail patients. There was no difference between the groups (RR: 0.95; 95% CI 0.85–1.06; *p* = 0.08; *I*^2^ = 52%; Supplementary File 4). There was also no difference in the use of renal replacement therapy between frail and non-frail patients in six of the 13 studies (RR: 1.07; 95% CI 0.76–1.51; *p* = 0.006; *I*^2^ = 69%; Supplementary File 5).

## Discussion

In this meta-analysis of 11 observational studies, we found that frailty was identified in approximately 40% of the elderly patients admitted to the ICU. We also found that frailty was associated with increased risks of short-term, in-hospital, and long-term mortality. In the retrospective studies, we found that frail patients were likely to have a prolonged hospital LOS. There was no significant difference between the frail and non-frail groups in the use of mechanical ventilation, use of vasoactive therapy, or the use of renal replacement therapy.

Our data showed that the prevalence of frailty in the elderly patients admitted to ICU was higher than that reported in a previous study (18), which included the adult ICU hospital population and was not specifically focused on the elderly patients. The VIP2 study (28) suggested that the prevalence of frailty was 40.2% in very old patients admitted to ICU, and our result appeared consistent with this finding. Meanwhile, most of the published data (40–43) showed that frail patients were likely to be more susceptible to coronavirus disease 2019 (COVID-19) with a prevalence of frailty as high as 51.1% (40). This result provided empirical evidence of the widely held belief that frail patients are relatively more susceptible to the pathogens.

Frailty was recognized initially in the field of geriatric medicine, and it has recently been increasingly identified as an essential determinant of prognosis in patients admitted to ICU. Our results were consistent with those of a previous study which showed that frail patients were at higher risk than non-frail patients of poor outcomes in different settings (44–46). The explanation for this finding involves the changes in pathophysiology in frail patients. Frail ICU patients have neuromuscular weakness, inflammation, and immunosenescence (47), which cause molecular and cellular deficits (48). These factors may increase susceptibility to pathogens in patients admitted to ICU. Furthermore, a diminished reserve in the multiple systems in frail patients might increase adverse outcomes such as mortality and the use of mechanical ventilation. Furthermore, frail patients have reduced resilience making their recovery more difficult (44) and prolonging their hospital LOS. In our meta-analysis, we found that elderly frail ICU patients had longer hospital LOSs, although this was only reported in the retrospective studies.

There was no significant difference between frail and non-frail patients in the use of mechanical ventilation, vasoactive agents, or renal replacement therapy, and we did not find a significant difference in hospital LOS in the prospective studies. The study by Heyland reported a higher rate of mechanical ventilation in non-frail patients than in frail patients (30). This result is unexpected because diminished resilience would be likely to increase the possibility of the need for advanced ICU support in frail patients. Critically ill frail patients may be more likely to receive mechanical ventilation due to decreased oxygen uptake and weakness. Furthermore, because of immunosenescence, it may take more time for critically ill frail patients to recover (47). During the COVID-19 epidemic, data (40) have shown that frail patients had prolonged hospital LOSs, which was not consistent with the data from the prospective studies in our meta-analysis. Possible explanations for these results are the limitations of medical care influenced by frailty, the incomplete reporting of data, the discharge pattern, and survival bias. Critically ill frail patients are likely to die earlier than non-frail patients, which may have reduced their hospital LOS and use of advanced organ support.

To reduce the heterogeneity due to the use of different methods to assess frailty, we performed a subgroup analysis according to the assessment method. The use of various methods to assess frailty should be considered. The FP (39) model and the cumulative deficit model (38) were developed to provide a theoretical framework for research on frailty. The FP model was first validated by Fried et al. The FP identifies frailty on the basis of five biological phenomena that result from the functional decline of multiple physiological systems (slow walking speed, low physical activity level, impaired grip strength, unintended weight loss, and self-reported exhaustion), while the cumulative deficit model calculates the FI on the basis of a range of health deficits (signs, symptoms, disabilities, impairments, and diseases). In our meta-analysis, two studies (26, 34) used the FI, and one study (31) used both the FP and CFS. To improve the ease of assessment of frailty in routine clinical practice, other tools have been developed, such as the CFS (37) and mFI. The CFS is an easy-to-use frailty measure with nine items with scores ranging from fit to terminally ill. The patients are considered to be frail when the CFS is more than 5 points. Eight studies included in our analysis used the CFS in the context of critical illness, enabling practitioners to rapidly screen for frailty, and the mFI (29) has also been validated in patients admitted to ICU. Future studies on the reliability, validity, and feasibility of frailty measures in the setting of the ICU are required.

There are several potential limitations of our meta-analysis. First, the included elderly patients had a range of underlying conditions; therefore, the prognostic significance of frailty in patients with acute respiratory distress syndrome, shock, and other types of organ failure should be confirmed in the future studies. Second, various frailty assessment tools were adopted in the included studies, leading to unavoidable heterogeneity. At last, it should be noted that the studies included in our meta-analysis were observational and may have been prone to bias.

## Conclusions

In conclusion, frailty is common in the elderly patients admitted to ICU, and it is associated with an increased risk of mortality. Furthermore, in the retrospective studies, elderly frail patients had a prolonged hospital LOS.

## Data Availability Statement

The original contributions presented in the study are included in the article/Supplementary Material, further inquiries can be directed to the corresponding author/s.

## Author Contributions

FX was responsible for the conception and design of the study, acquisition, the analysis and interpretation of the data, the drafting and revision of the article, and gave final approval of the version to be published. JZ and SM were responsible for the acquisition and analysis of data. HQ participated in the data analysis and the interpretation of the results. FG was responsible for the conception and design of the study, the analysis and interpretation of the data, the drafting and revision of the article, and gave final approval of the version to be published. All authors read and approved the manuscript.

## Funding

This work was supported by a grant from the National Natural Science Foundation of China (Grant Number: 6590000262) and the Natural Science Foundation of Jiangsu Province (SBK2020041068).

## Conflict of Interest

The authors declare that the research was conducted in the absence of any commercial or financial relationships that could be construed as a potential conflict of interest.

## Publisher's Note

All claims expressed in this article are solely those of the authors and do not necessarily represent those of their affiliated organizations, or those of the publisher, the editors and the reviewers. Any product that may be evaluated in this article, or claim that may be made by its manufacturer, is not guaranteed or endorsed by the publisher.

## Supplementary Material

Supplementary File 1 Quality assessment of included studies with the Newcastle–Ottawa Scale.

Supplementary File 2 The association of frailty with the hospital length of stay in elderly patients admitted to ICU.

Supplementary File 3 The association of frailty with the use of mechanical ventilation in elderly patients admitted to ICU.

Supplementary File 4 The association of frailty with the use of vasoactive therapy in elderly patients admitted to ICU.

Supplementary File 5 The association of frailty with the use of renal replacement therapy in elderly patients admitted to ICU.
