# Supplementary material for: Association of Frailty With the Risk of Mortality and Resource Utilization in Elderly Patients in Intensive Care Units: A Meta-Analysis
Source: Front Med (Lausanne). 2021 Oct 4;8:637446. doi: 10.3389/fmed.2021.637446 (PMC8521007; doi:10.3389/fmed.2021.637446)
Supplement: Supplementary File 1 — Quality assessment of included studies with the Newcastle–Ottawa Scale. [file Table_1.DOC]

**Additional file 1: Quality Assessment of Included Studies by** **Newcastle–Ottawa Scales**

| **Study** | **Selection** | | | | **Comparability** | **Outcome** | | | **Total**  **Score** |
| --- | --- | --- | --- | --- | --- | --- | --- | --- | --- |
|  | **Exposed**  **Cohort** | **Nonexposed**  **Cohort** | **Ascertainment**  **of Exposure** | **Outcome**  **of Interest** |  | **Assessment**  **of Outcome** | **Length of**  **Follow-up** | **Adequacy**  **of Follow-up** |  |
| Darvall 2019 | * | * | * | * | * | * | * | * | 8 |
| Fernando 2019 | * | * | * | * | ** | * | * | * | 9 |
| Ferrante 2018 | * | * | * | * | ** | * | * | * | 9 |
| Flaatten 2017 | * | * | * | * | ** | * | * | * | 9 |
| Guidet 2019 | * | * | * | * | ** | * | * | * | 9 |
| Hamidi 2019 | * | * | * | * | - | * | - | * | 6 |
| Heyland 2015 | * | * | * | * | ** | * | * | * | 9 |
| Jung 2021 | * | * | * | * | ** | * | - | * | 8 |
| Le Maguet 2014 | * | * | * | * | ** | * | * | * | 9 |
| López 2019 | * | * | * | * | - | * | - | * | 6 |
| Pasin 2020 | * | * | * | * | ** | * | * | * | 9 |
| Silva-Obregón 2020 | * | * | * | * | - | * | - | * | 6 |
| Zeng 2015 | * | * | * | * | * | * | * | * | 7 |
