# Supplementary figures and images for: Association of Frailty With the Risk of Mortality and Resource Utilization in Elderly Patients in Intensive Care Units: A Meta-Analysis
Source: Front Med (Lausanne). 2021 Oct 4;8:637446. doi: 10.3389/fmed.2021.637446 (PMC8521007; doi:10.3389/fmed.2021.637446)

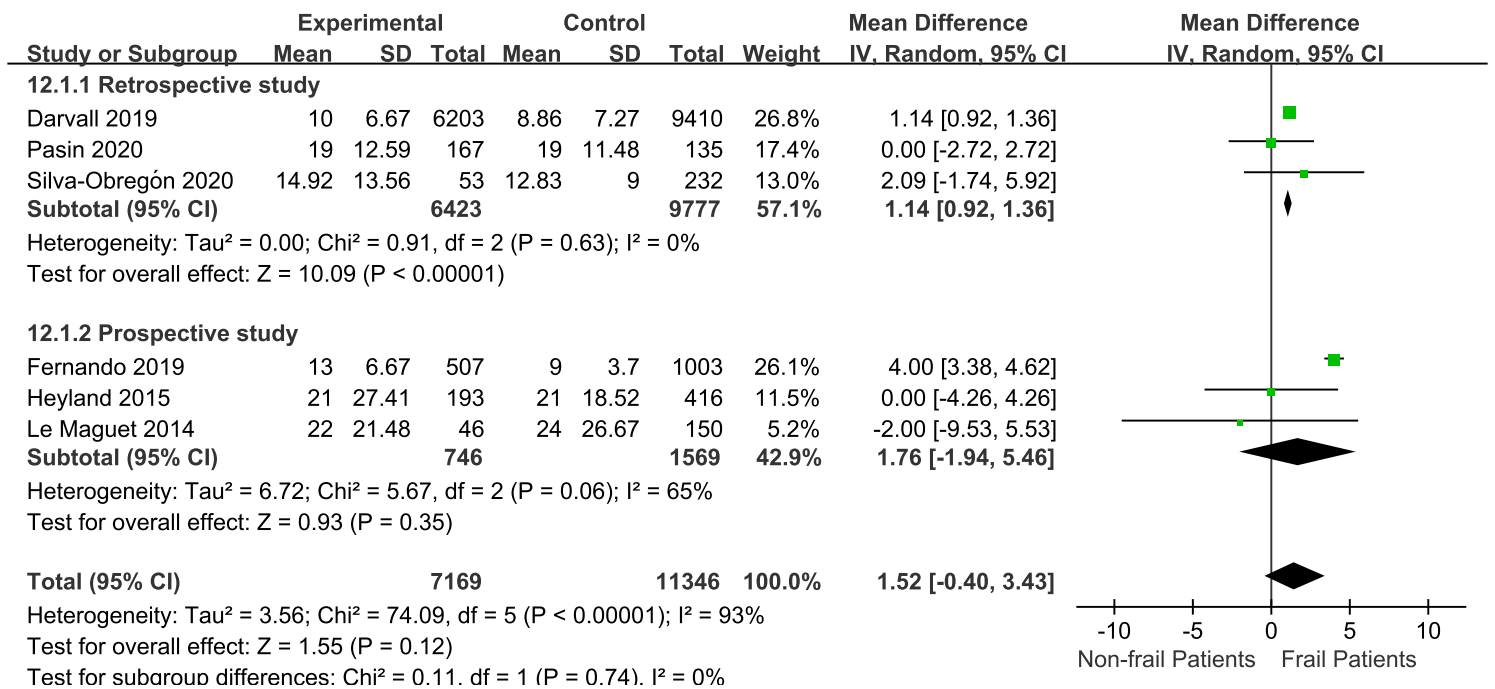

Supplement: Supplementary File 2 — The association of frailty with the hospital length of stay in elderly patients admitted to ICU. [file Data_Sheet_1.PDF]

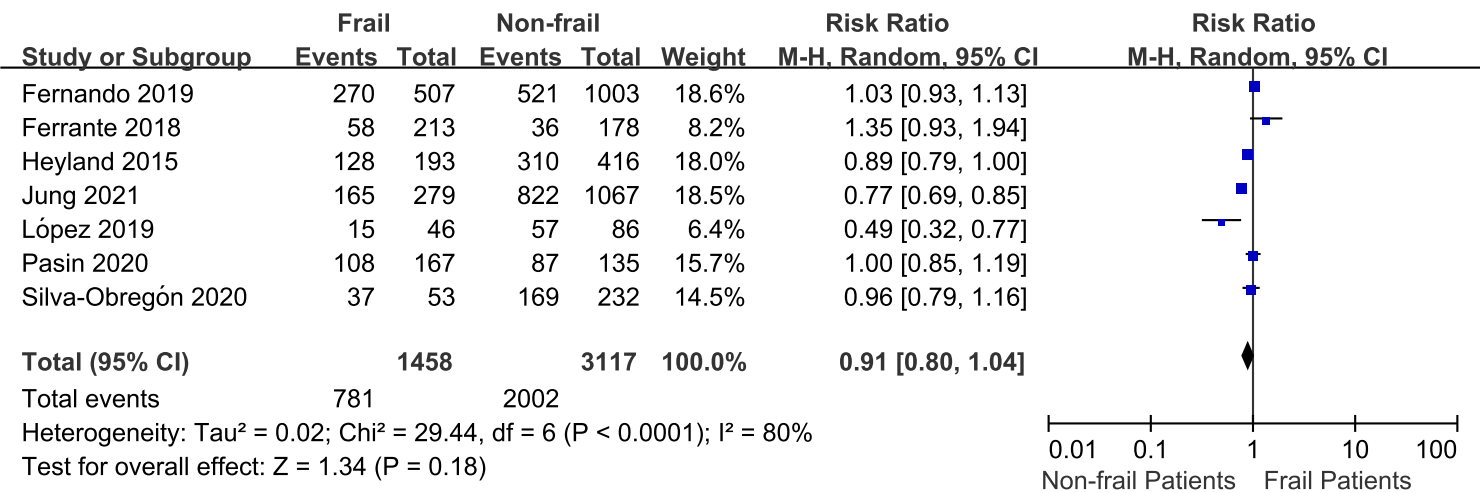

Supplement: Supplementary File 3 — The association of frailty with the use of mechanical ventilation in elderly patients admitted to ICU. [file Data_Sheet_2.PDF]

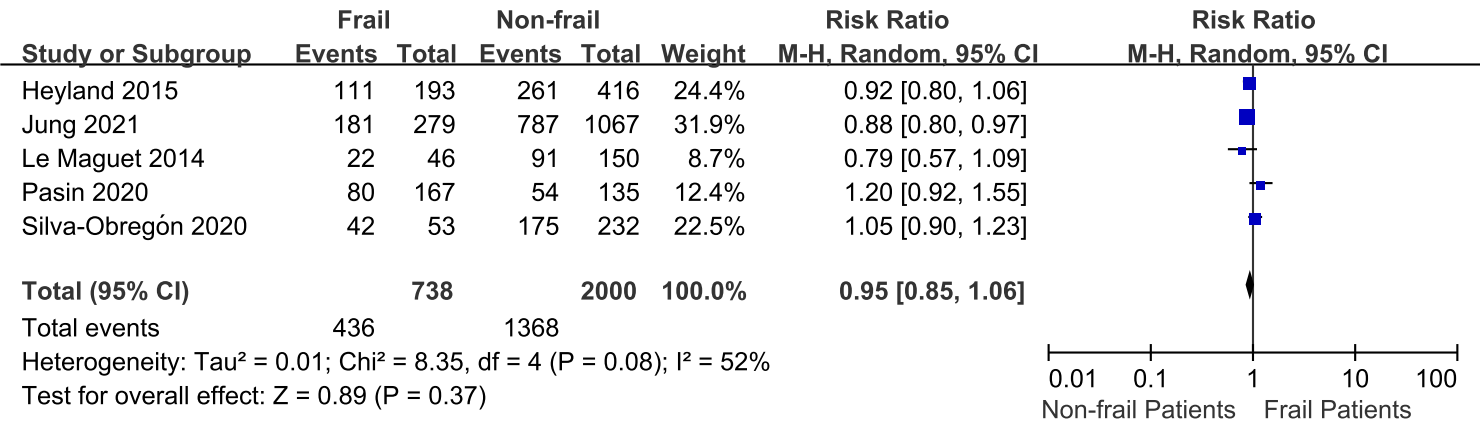

Supplement: Supplementary File 4 — The association of frailty with the use of vasoactive therapy in elderly patients admitted to ICU. [file Data_Sheet_3.PDF]

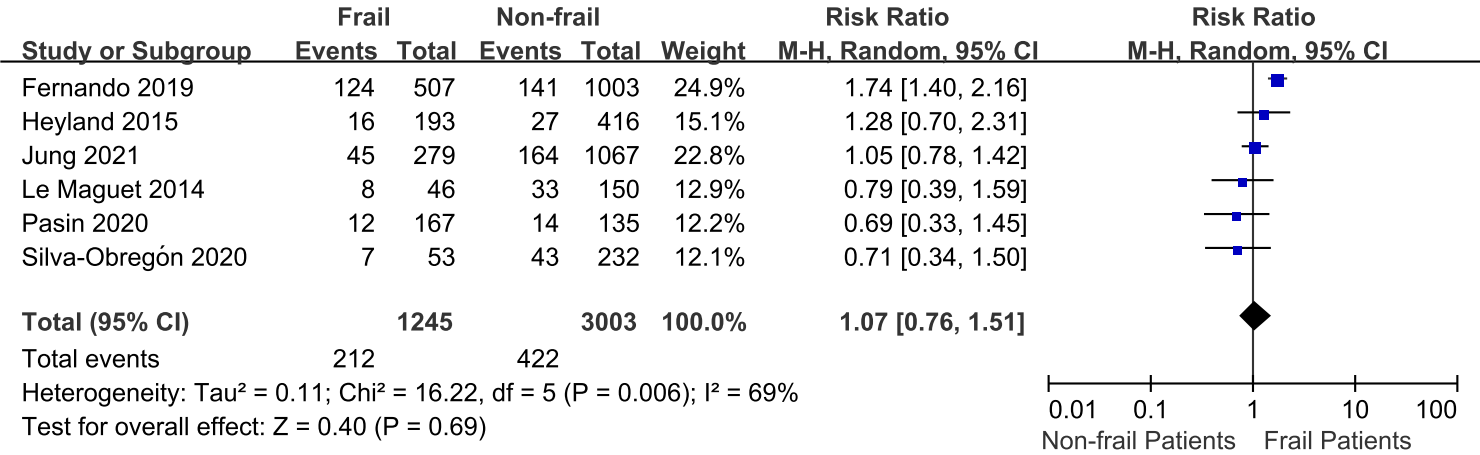

Supplement: Supplementary File 5 — The association of frailty with the use of renal replacement therapy in elderly patients admitted to ICU. [file Data_Sheet_4.PDF]
